# Effects of 8 weeks of Stealth^® ^supplementation on body composition, muscle strength and mass, markers of satellite cell activation, and clinical safety markers in males

**DOI:** 10.1186/1550-2783-12-S1-P9

**Published:** 2015-09-21

**Authors:** Micheil B Spillane, Neil A Schwarz, Darryn S Willoughby

**Affiliations:** 1 Health, Physical Education and Leisure Studies, University of South Alabama, Mobile, AL 36688, USA; 2 Exercise and Biochemical Nutrition Lab, Department of Health, Human Performance, and Recreation, Baylor University, Waco, TX 76798, USA

## Purposes

This study determined the effect of 8 weeks of heavy resistance exercise combined with oral ingestion of either a placebo or Stealth^® ^dietary supplement on body composition, muscle strength and mass, hemodynamics, myofibrillar protein content, serum (IGF-1, HGF, GH), muscle total DNA content, c-Met, and the myogenic regulatory factors (Myo-D, Myogenin, MRF-4).

## Methods

Twenty non-resistance-trained males were randomly matched by age and body mass in a double-blind fashion being assigned to either a placebo (maltodextrose) or Stealth^® ^group. Testing was conducted at baseline (day 0) followed by 8 weeks of a periodized 4-day per week resistance training program of 3 × 10 reps at 70-80% of their 1-RM. The program was split into two upper and two lower extremity workouts per week with post testing occurring at (day 57). Both groups consumed 2 servings (312g) (1248 kcals) per day. During exercise sessions the placebo group consumed (156g) of maltodextrose 30 min before and after exercise. The Stealth^® ^group consumed (22g fat, 158g carbohydrates, 94g protein). During non-training days both groups consumed the 2 servings in the morning upon waking. Both the placebo and Stealth^® ^groups consumed an isocaloric diet (~2500 kcals) and the additional (1,248 kcals) for a total of (~3750 kcals) each day. Data were analyzed with separate 2 × 2 factorial analyses of variance (ANOVA) with repeated measures (p < 0.05).

## Results

For dietary intake, there were no significant differences in total calories (p = 0.346), protein (p = 0.689), and fat (p = 0.275) between testing sessions. A significant difference in carbohydrate (p = 0.003) between testing session was shown, but no difference (p = 0.737) between groups was observed. Hemodynamic measurement between testing session for resting heart rate (p = 0.208) and SBP (p = 0.192) were not significant between testing sessions. However, DBP (p = 0.047) was significant but no differences (p = 0.686) between groups were observed. A significant increase in body mass (p = 0.001), body water (p = 0.001), body fat % (p = 0.001), and fat mass (p = 0.001) were shown between testing sessions. Only body water was significantly (p = 0.030) greater within the stealth^® ^group. No significant difference in fat free mass (p = 0.068) was shown between testing session for either group. A significant difference in upper body strength (p = 0.024) and lower body strength (p = 0.001) was shown between testing sessions for both groups. However, no significant difference between upper body (p = 0.989) and lower body (p = 0.097) strength was observed between the supplement groups. Serum IGF-1 (p = 0.270), HGF (p = 0.070), and GH (p = 0.397) were not significantly different between testing sessions. No significant difference between testing sessions for myofibrillar protein (p = 0.108), total DNA (p = 0.217), Myo-D (p = 0.093), and Myogenin (p = 0.070) were observed. A significant difference between testing session in c-MET (p = 0.023) and MRF-4 (p = 0.044) were shown. Only the placebo (p = 0.047) group was < Stealth^® ^for c-Met.

## Conclusions

Heavy resistance training with a high caloric proprietary blend weight gain dietary supplement does not improve markers for skeletal muscle hypertrophy. Significant increases in body mass, fat mass and body fat % were shown for both placebo and Stealth^®^.

